# A scoping review of the risk factors and strategies followed for the prevention of COVID-19 and other infectious diseases during sports mass gatherings: Recommendations for future FIFA World Cups

**DOI:** 10.3389/fpubh.2022.1078834

**Published:** 2023-01-16

**Authors:** Nour Waleed Zuhair Alhussaini, Usra Abushara Mohamed Elshaikh, Noor Ahmed Hamad, Maisa Ayman Nazzal, Manal Abuzayed, Ghadir Fakhri Al-Jayyousi

**Affiliations:** ^1^Department of Public Health, College of Health Sciences, QU Health, Qatar University, Doha, Qatar; ^2^Department of Infection Control Unit, Ibn Sina Specialized Hospital, West Bank, Palestine; ^3^Health Promotion Division, Department of Public Health, Ministry of Public Health, Doha, Qatar

**Keywords:** world cup, COVID-19, infectious diseases, sports events, mass gatherings, prevention, Qatar

## Abstract

**Objective:**

Sports mass gatherings of people pose particular concerns and place an additional burden on the host countries and the countries of origin of the travelers. It is imperative to identify how countries dealt with various communicable diseases in the context of previous world cups and identify possible advice for protection from outbreaks.

**Methods:**

A scoping review was employed in this study and a PRISMA extension for scoping reviews was employed to guide the reporting of this study. A systematic search was performed using PubMed, Embase, Web of Science, SCOPUS, SportDiscus, and Google scholar. The search strategy included two main strings viz “communicable disease” AND “sport” AND “setting” as keywords for each string. A total of 34 studies were included in this review.

**Results:**

Information on risk factors for infectious diseases during FIFA, and recommendations for disease prevention in various stages of the event: pre-event, during, and post-event were charted. These strategies can be achieved with the empowerment of the public by enhancing their social responsibility and the coordination between the healthcare system, the ministry of public health, and other stakeholders.

**Conclusion:**

The findings will support planning for protection strategies to prevent any outbreak while having the FIFA World Cup or any other sports gatherings. A model was constructed to present the findings and recommendations from this review.

## 1. Introduction

Mass gatherings of people pose particular concerns and place an additional burden on the host countries and the countries of origin of the travelers (1). These mass gatherings could range from global sporting events to global religious occurrences (2–5). A number of health concerns can accompany mass gatherings, including increased human crowding and the spread of pathogens, which can raise the chance of infectious disease spread among attendees, specifically respiratory disease infections, causing pandemics (6, 7).

The World Cup is one of the world's biggest events bringing people and countries together in celebration and competition. The World Cup was hosted previously by several countries where different strategies to reduce the risk and the impact of acquiring communicable diseases during a mass gathering were implemented. Strategies have focused on pre-travel consultation (8, 9), the provision of standard operating procedures for epidemic response (10), and enhanced international multi-disciplinary surveillance to monitor and assess the risk of any infectious disease threats and promptly detect incidents (11–16). Medical facilities were established at the airport for the isolation of patients and extensive staff training was conducted in the use of infection control practices (14). Additionally, close Devi Priya rapid detection of infectious diseases (14).

### 1.1. FIFA World Cup 2022

Qatar served as the first Middle Eastern host of the FIFA World Cup in 2022 (17) located on the western coast of the Arabian Gulf (18). The Qatar FIFA World Cup 2022 welcomed 32 teams and was hosted across eight stadiums (19). Stadiums were constructed as some of the most eco-friendly and architecturally innovative stadiums with cooling technology capable of reducing temperatures within it by up to 20°C (36°F) (19). Qatar is home to around 3 million people (18) from around the world and approximately welcomed external fans equal to more than half of the country's total population. Certainly, hosting with such an enormous number of fans like the FIFA World Cup necessitate vigorous security measures to protect players, spectators, and residents.

The World Cup 2022 came with exceptional challenges being held against the background of Corona virus disease (COVID-19) pandemic. Qatar has one of the lowest COVID-19 mortality rates in the world. This could be due to the government's quick and comprehensive measures, which include adjusting public health measures based on the ongoing epidemiological surveillance system, strategic testing, COVID-19 awareness campaigns, and free vaccinations to the public (20). The government has also implemented strict travel regulations for individuals coming from abroad (21). However, other infectious diseases and resulting epidemics had become significant health threats around the time of this event, such as monkeypox (22) and Middle East respiratory syndrome coronavirus (MERS-CoV) (23). The risk of an outbreak of different infections would be even more significant in such events as the visitors were expected to be from more diverse backgrounds. In order to reduce the risk of communicable disease outbreaks during the World Cup, event planners in Qatar recommended conducting a thorough risk assessment prior to the event, and creating risk management/communication plans (24).

The literature reported that large sport events and other mass gatherings impose a risk in increasing the cases of COVID-19 and other infectious diseases (25, 26). No studies have reviewed the health risks and prevention of infectious diseases during sport mass gathering events; therefore, it needs to be thoroughly reviewed in order to be able to develop effective risk management/communication plans. This scoping review will map the available literature regarding the risk factors of infectious diseases including COVID-19 and strategies followed for prevention in previous world cups or sports events to provide recommendations for future FIFA World Cups and other sports mass gatherings.

## 2. Methods

This review was conducted by employing the Preferred Reporting Items for Systematic Reviews and Meta-Analyses extension for Scoping Reviews (PRISMA-ScR) (27). During the review, we followed these steps: Identifying the research question, identifying the relevant studies, selecting the studies, extracting the data, collating, summarizing, and synthesizing the results.

### 2.1. Identifying the research question

The overarching research question for our review is: what are the strategies followed for the prevention of infectious diseases in sports mass gatherings? To address the main question, we also identified the following specific questions:

1. What are the risk factors for infectious diseases from previous world cups or sports events with mass gatherings?
2. What are the recommended strategies to be followed for the prevention of infectious diseases before, during, and after the sports mass gatherings?

### 2.2. Identifying the relevant studies

A systemic search was performed using PubMed, Embase, Web of Science, SCOPUS, SportDiscus, and Google scholar. These databases were searched without restrictions to reclaim any publications related to our research question in the period between 2010 and 24 January 2022. The search strategy included three main strings viz “communicable disease” AND “sport” AND “setting” as keywords for each string were used when building the search strategy. The combination of keywords used were (world cup OR sport OR stadium) AND (infectious disease OR communicable disease OR virus) AND (mass gather OR crowd). Table 1 describes the search strategies used to gather the articles from the mentioned databases. To assure not missing any publications related to our purpose, google scholar was further searched, and the reference lists of the selected articles were also screened for articles that might have been not captured from the initial search of the databases.

**Table 1:** Search strategies.

**Database**	**Search strategy**	**Number of studies**
Pubmed	#1 ((“world cup”[Title/Abstract]) OR (stadium^*^[Title/Abstract])) OR (sport^*^[Title/Abstract]) #2 ((“infectious disease^*^”[Title/Abstract]) OR (“communicable disease^*^”[Title/Abstract])) OR (virus^*^[Title/Abstract]) #3 (“mass gather^*^”[Title/Abstract]) OR (crowd^*^[Title/Abstract]) ((#1) AND (#2)) AND (#3)	36
Embase	#1 “world cup:”ab,ti OR sport^*^:ab,ti OR stadium^*^:ab,ti #2 “infectious disease^*^:”ab,ti OR “communicable disease^*^:”ab,ti OR virus^*^:ab,ti #3 “mass gather^*^:”ab,ti OR crowd^*^:ab,ti #1 AND #2 AND #3	25
Web of science	#1 ((TS = (“world cup”)) OR TS = (sport^*^)) OR TS = (stadium^*^) #2 ((TS = (“infectious disease^*^”)) OR TS = (“communicable disease^*^”)) OR TS = (virus^*^) #3 (TS = (“mass gather^*^”)) OR TS = (crowd^*^) #1 AND #2 AND #3	47
SPORTDiscus	(“world cup” OR stadium^*^ OR sport^*^) AND (“infectious disease^*^” OR “communicable disease^*^” OR virus^*^) AND (“mass gather^*^” OR crowd^*^)	6
SCOPUS	(TITLE-ABS (“world cup”) OR TITLE-ABS (sport^*^) OR TITLE-ABS (stadium^*^)) AND (TITLE-ABS (“infectious disease^*^”) OR TITLE-ABS (“communicable disease^*^”) OR TITLE-ABS (virus^*^)) AND (TITLE-ABS (“mass gather^*^”) OR TITLE-ABS (crowd^*^))	44

### 2.3. Selecting studies

Any study investigating infectious diseases in the previous FIFA World Cups and other sporting events with mass gatherings was eligible to be included. The first two authors and the corresponding author independently screened titles and abstracts of the citations retrieved from the search. Then, these articles were divided among the same three authors to assess full texts of the relevant records independently. During the study selection and assessment process, the three authors would meet to resolve any conflict and reach an agreement.

Inclusion criteria:

- Articles covering sports mass gatherings.
- Peer-reviewed articles published in the period between 2010 and 24 January 2022.
- Articles covering other mass gatherings, but/or with recommendations for infectious disease prevention in sports events.
- Articles addressing risk factors of viral infectious diseases and/or protection and prevention of infectious diseases.
- Review articles providing recommendations for infectious disease prevention in sports events and other mass gatherings.
- Articles on infections that are transmitted through air, direct contact, or droplets.
- Articles published in English.

Exclusion criteria:

- Small-scale sports events.
- Articles addressing risk factors irrelevant to viral infectious diseases.
- Articles addressing infectious diseases caused by pathogens other than viruses.
- Reports, book chapters, and conference papers.
- Articles on vector-borne disease.

### 2.4. Extracting the data

A priori identified spreadsheet was developed for data extraction. Data were reported in two tables: Table 1 reported the characteristics of the included studies and Table 2 reported the strategies and recommendations for infectious disease prevention pre, during, and post sports mass gatherings events. Specifically, the following information was included in Table 1: the first author of the study, publication year, country, article type, setting of the study, population description, type of sport, and type of infection. While the second table included the following information: risk factors of infectious diseases, strategies that are recommended to be followed before, during, and after the sports mass gatherings to prevent infectious diseases, and general recommendation for infectious disease prevention in sports mass gatherings.

**Table 2:** Characteristics of included studies.

**References**	**Country**	**Article type**	**Setting of research study**	**Population description**	**Type of sport**	**Type of infection**
Blumberg et al. (8)	South Africa	Editorial	2010 FIFA World Cup in South Africa	Populations in mass gatherings	Football	H1N1, H3N2, HIV, malaria, food borne illnesses
Gallego et al. (10)	Brazil	Scoping review	2014 FIFA World Cup in Brazil	Populations in mass gatherings	Football	Yellow fever, dengue, chikungunya fever, chagas disease, malaria, leishmaniasis, cutaneous larva migrans, rickettsiosis, tuberculosis, influenza, hantavirus, leptospirosis, schistosomiasis, HIV/AIDS, foodborne illnesses
Dove et al. (28)	NS	Scoping review	Sport events	Athletes	Multiple sports	COVID-19
Parnell et al. (29)	NS	Commentary	Mass gatherings in general	Populations in mass gatherings	NS	COVID-19
Mantero et al. (30)	South Africa	Scoping review	2010 FIFA World Cup in South Africa	Populations in mass gatherings	Football	Influenza, measles
Griffith et al. (11)	Japan	Summary of national surveillance data	• 2019 Rugby world cup • 2020 Tokyo summer olympic and paralympic games	Travelers	Multiple sports	Rubella, invasive pneumococcal disease, measles, non-A and non-E viral Hepatitis, hepatitis A, Invasive hemophilus influenzae disease, tetanus, typhoid fever, invasive meningococcal disease, Japanese encephalitis, influenza, varicella, mumps, pertussis
Annear et al. (31)	Japan	Scoping review	2020 Tokyo summer olympic and paralympic games	Athlete and spectator	Multiple sports	Mumps, measles, chicken pox, H1N1
Miles and Shipway (32)	N/A	Scoping review	Sport events	Tourists, travelers, athletes	Multiple sports	COVID-19
Alshahrani et al. (12)	Qatar	Scoping review	FIFA World Cup 2022	Populations in mass gatherings	Football	Influenza, COVID-19, hepatitis A
Pshenichnaya et al. (9)	Russia	Editorial	World cup Russia 2018	Attendees	Football	Influenza, tuberculosis, rabies, west nile fever, gastrointestinal infections, measles, mumps, tick-borne encephalitis (TBE), lyme disease
Ahmed and Memish (33)	NS	Scoping review	Hajj and sporting events (olympic games)	Populations in mass gatherings	Multiple sports	Biological agents (terrorism) hepatitis A
Abubakar et al. (34)	NS	Series (report)	Hajj and sporting events (olympic games, cricket worldwide, world cups)	Populations in mass gatherings	Multiple sports	Multiple infectious diseases (NS)
Gaines et al. (35)	Brazil	Special communication	• 2014 FIFA World Cup in Brazil • 2016 summer olympic and paralympic games in Brazil	Populations in mass gatherings	Multiple sports	Hepatitis A, hepatitis B, yellow fever, rabies, dengue
Wilson and Chen (13)	Brazil	Editorial	• 2014 FIFA World Cup in Brazil • 2016 summer olympic and paralympic games in Brazil	Travelers	Multiple sports	Influenza, measles, chikungunya
Blumberg et al. (36)	West Africa	NS	• African youth games, 2014 • Africa cup of nations, equatorial guinea, 2015 • All-Africa games, Republic of Congo, 2015	Populations in mass gatherings	Multiple sports	Ebola virus
Wilson et al. (14)	Brazil	Cross-sectional study	2014 FIFA World Cup and the 2016 summer olympics	Populations in mass gatherings	Multiple sports	Dermatologic problems, diarrhea, febrile systemic infections, dengue, and malaria
Wong et al. (15)	Hong Kong	Randomized controlled study	Hong Kong premier league (HKP)	Football players	Football	COVID-19
Duarte Muñoz and Meyer (37)	NS	Editorial	Mass gatherings in general	Populations in mass gatherings	Football	COVID-19
Hoang and Gautret (38)	NS	Scoping review	• The summer and winter olympics • FIFA World Cup and the EURO football cup from 1984 through 2015	Populations in mass gatherings	NS	Measles, Influenza, Gastrointestinal infections, and respiratory infections
Vyklyuk et al. (39)	NS	NS	Mass gatherings in general	Populations in mass gatherings	NS	COVID-19
Gautret et al. (40)	Brazil and Korea	Cross-sectional study	• The 2016 summer olympic and paralympic games in Brazil • The 2018 winter olympics in south Korea	Ill travelers	NS	NS
Al-Romaihi et al. (41)	Qatar	Cross-sectional study	Mass gatherings in general	Populations in mass gatherings	NS	NS
McCloskey et al. (16)	London	Series	London 2012 olympic and paralympic games	Populations in mass gatherings	Multiple sports	Multiple (NS)
McCloskey et al. (42)		Comment	Mass gatherings in general	Populations in mass gatherings	NS	COVID-19
Murray et al. (43)	United states	Report	Major league baseball	Team members	Baseball	COVID-19
Chan et al. (44)	Australia	Case study	Sport league	Populations in mass gatherings	Football	COVID-19
Aitsi-Selmi et al. (45)	NA	Scoping review	Mass gatherings in general	Populations in mass gatherings	NS	NS
Drury et al. (46)	UK	Scoping review	Live events: sports and music arena events	Populations in mass gatherings	NS	COVID-19
Al-Tawfiq et al. (47)	Saudi Arabia	Scoping review	Hajj pilgrimage	Pilgrims	NS	Influenza, Rhinovirus
Dénes et al. (48)	Ukraine	Epi study	2012 UEFA European football championship	Populations in mass gatherings	Football	Measles
Leal Neto et al. (49)	Brazil	Participatory surveillance	2014 FIFA World Cup in Brazil	Healthy cup app users	NS	Influenza, measles, rubella, cholera, acute diarrhea, dengue fever
Chiampas and Ibiebele (50)	NS	Scoping review	Sports and mass gatherings in general	Athletes, travelers, audience	NS	COVID-19
Hassanzadeh-Rad and Farzin (51)	NS	Letter to editor	Sports	Populations in mass gatherings	NS	COVID-19
Eberhardt et al. (52)	Brazil	Prospective case-control survey	2014 FIFA World Cup in Brazil	Travelers	NS	NS

NS, Not specified; N/A, not applicable.

### 2.5. Synthesizing the results

In our review, we clustered the infectious disease prevention strategies into three stages: pre-event, during the event, and post- sports event mass gatherings. Description of the scope of literature was presented in a model according to the various strategies and recommendations followed to prevent infectious diseases in various stages of the planning, implementation, and follow-up after the sports mass gatherings are finalized. The model also showed how these strategies of the three stages are impeded in different three contexts that would support their implementation, monitoring and evaluation. These contexts are community/ public social responsibility, preparedness of the health care system, and the regulations/policy/guidelines of public health authorities and other partners.

## 3. Results

**Figure 1:**
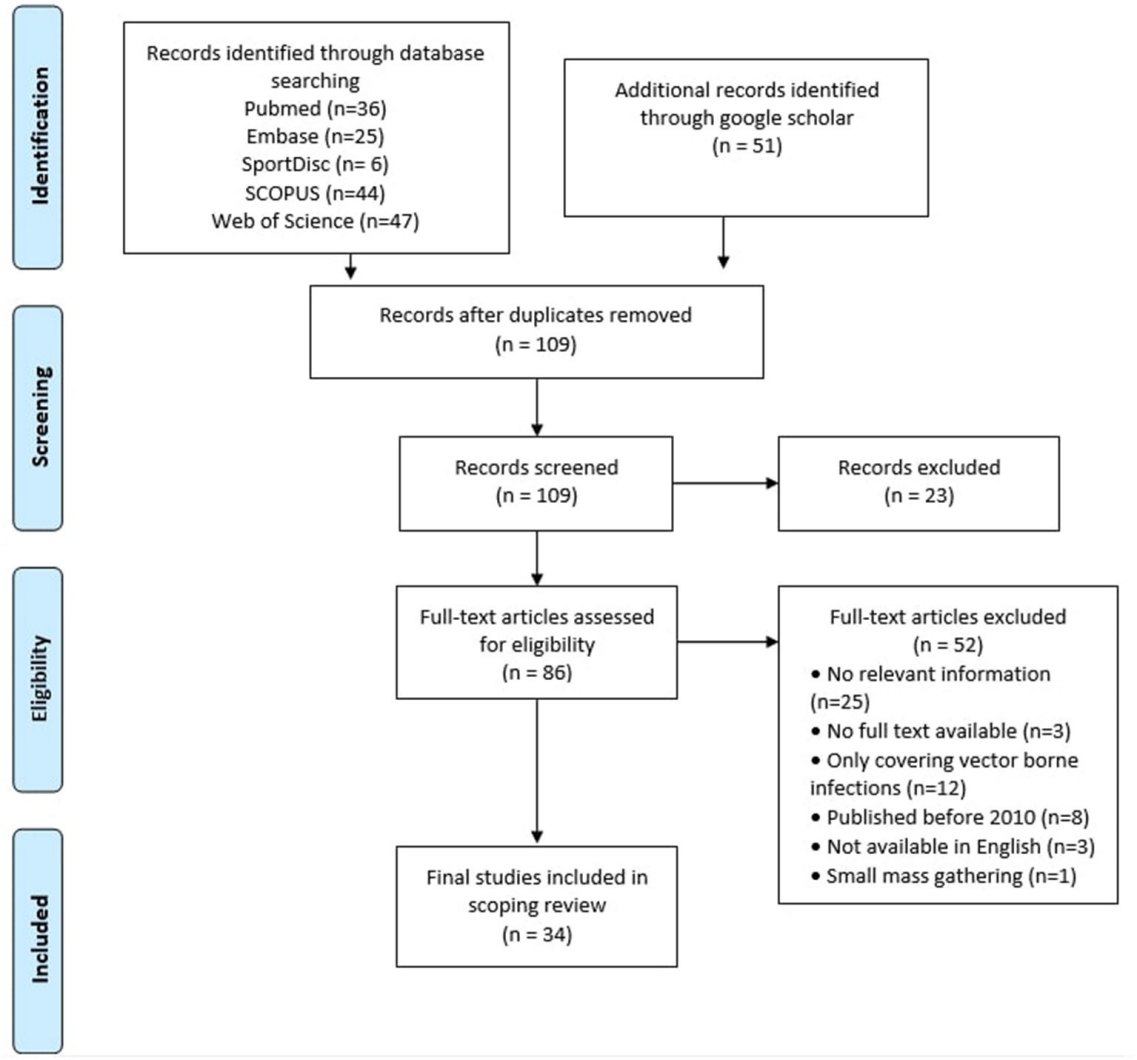
PRISMA flow chart for recording included studies.

Following the mentioned search strategy, 158 records were retrieved from the mentioned databases search. The remaining records, after removing the duplicates, amounted to 109 records. After screening the titles and abstracts, 23 were excluded; the remaining 86 full-text articles were assessed for eligibility, and 34 studies were reserved for this review. The PRISMA diagram demonstrates the selection process and clarifies the reasons for exclusion of other studies (Figure 1).

### 3.1. Characteristics of the included studies

The studies were divided by design into 11 scoping reviews (10, 12, 28, 30–33, 38, 45–47, 50), three cross-sectional studies (14, 40, 41), four editorials (8, 9, 13, 37), two series reports (16, 34), one randomized controlled study (15), one commentary (29), one summary of national surveillance data (11), one special communication (35), one comment (42), one report (43), one case study (44), one epidemiological study (48), one participatory surveillance (49), one letter to the editor (51), one prospective case-control survey (52), one epidemiological study (48), and two articles were not specified (36, 39). The majority of these studies reported their findings from one country, one study reported data from two countries, and 12 studies were not in specific countries. In 23 studies, the settings of the research were related to sports events, six studies looked at mass gatherings in general, four studies looked at two settings:

Hajj and sporting events or music arena events or mass gatherings in general and one study looked at the Hajj pilgrimage. The majority of studies have described populations in mass gatherings, and the rest of the studies have described athletes or/and travelers or/and the audience. In 11 studies, the types of sports that were described were multiple sports, in nine studies it was football, in one study it was baseball, while in 13 studies the type of sport was not specified. The type of infection that was addressed varied between studies. In 15 studies, they described more than one infection. The most common infection addressed in the majority of these studies was influenza and 12 studies were focused on COVID-19 solely. In six studies, there was no specific type of infection, while two studies described the Ebola virus or measles (see Table 2 for the characteristics of the included studies).

### 3.2. Risk factors of the infectious diseases' outbreaks in the included studies

In order to prevent the spread of infectious diseases through a specific event, several risk factors must be considered. In general, risks to travelers involve locally endemic infections that are unfamiliar to many travelers, or infections that are more likely to arise as a result of crowding related to mass events. In terms of risks to citizens, travelers carry pathogens that could initiate a local epidemic, such as the corona virus as well as influenza virus (13). Others included non-compliance with basic infection control and prevention standards such as poor hygiene, lack of sanitation, inadequate vaccination coverage, lack of immunity due to non-vaccination such as the fact that the majority of people who became ill with measles had not been vaccinated, close contact between the players, also the infection risky behaviors including touching the face and spitting (9, 12, 15, 28, 33, 38). Traveling is one of the most important factors in disease transmission particularly when visiting high-risk areas (10–13, 28, 29, 40), because the risk of disease transmission is across communities due to overcrowding, localized high population density (9, 12, 30, 34) and challenges in contact tracing due to the mobility of attendees with communicable diseases (8, 30). In addition to group identity, physical setting, climate, population participating in the event, and potential infections, crowd behaviors at live events may be affected by behaviors before and during the pandemic (34, 46), a study has also shown that transmission is most intense from February to June, with influenza peaking in June and July (14). In addition, one of the most critical factors is the ability to respond effectively and quickly to outbreaks and other emergency situations (41).

### 3.3. Pre-event strategies

In our review, several studies documented the pre-event preparations in worldwide sports events during epidemics. The immunization and vaccination for hepatitis A&B, yellow fever, rabies, mumps, measles, rubella, and influenza were recommended strategies, in which travelers should be encouraged to visit a health-care provider 4–6 weeks before travel to manage any risk through vaccinations (8, 9, 11–14, 31, 34–38, 47, 48, 50). Other strategies included educational messaging through targeted media and communications prior to the matches (8, 44), infection control practices such as hand hygiene, cough etiquette (8, 31, 36, 38, 44), pre-travel consultation (8, 10, 12, 52), and advice on the correct timing and use of personal protection measures (10, 36), self-isolation and quarantine for new arrivals or symptomatic individuals (31), physical distancing measures and regular COVID-19 testing were proposed with strict adherence required from staff, players, coaches, and others (28, 44), as well as travel precautionary measures including COVID-19 test certificates, quarantine, digital apps (12, 44), and travel restrictions by reducing flights and public transport (29).

The recommendations for the athletes, staff, and others included testing all athletic activities including pre-events, training sessions in the recognition and management of communicable diseases (12, 28, 36, 41), informing the travelers about their role in transmitting or preventing the transmission of the disease (11), as well as providing health education psychology supporting materials for athletes. Another study recommended that employees could operate from home to avoid having direct contact with athletes (15). Some studies mentioned the contribution of efforts to create multidisciplinary surveillance (40), and public health risk assessment to follow the principles of risk analysis, surveillance, and reporting in order to enhance public awareness of public health concerns (16, 42, 52), and how to inform about the health situation and any relevant advice and recommended actions (42). See Table 3 for summary of the pre-event strategies.

**Table 3:** Strategies pre, during, and post-events to prevent the spread of infectious diseases.

**References**	**Risk factors**	**Recommendations**			
		**Pre-event**	**During event**	**After event**	**General recommendations**
Blumberg et al. (8)	Mobility of attendees with communicable disease	– Immunity (vaccines) – Pre-travel consultation – Educational messaging (cough etiquette and hand hygiene	Availability of tissues and facilities to cleanse hands in public areas, voluntary isolation of mild cases at home when showing symptoms		Enhanced epidemic intelligence
Gallego et al. (10)	Visiting elevated risk areas possibility of travelers bringing the virus with them	– Pre-travel consultation – Advice on the correct timing and use of personal protection measures			– Enhanced epidemic intelligence to promptly detect incidents – Provision of standard operating procedures for epidemic response
Dove et al. (28)	– Close contact between football and soccer players – Travel also increases the risk of viral spread	Testing all athletic activities including pre-events	– Daily self-health checks – Universal masking on all sidelines – Testing athletic activities during the events	Testing athletic activities post-events	
Parnell et al. (29)	Travel is one of the key contributors to disease transmission	Travel restrictions, including reduced flights and public transport and route restrictions without compromising essential services	Use of social distancing measures		Community mitigation strategies
Mantero et al. (30)	Localized high population density, risk of importation of non-endemic diseases, exportation of endemic diseases, challenges in contact tracing due to visitor mobility, and temporary structures such as mass catering and accommodation for visitors		Adapted routine epidemic intelligence activities by the ECDC and was further enhanced by using a targeted and systematic screening approach through tailored tools (MediSys)		
Griffith et al. (11)	Traveling	– Up-to-date vaccinations with additional preventive measures should be included in pre-travel advice – Prioritized rubella, mumps, and influenza for pre-travel advice – Travel advisers should also consider individual traveler behaviors and itineraries. Health professionals should also inform travelers about the role they could play in transmitting or preventing the transmission of disease to MG attendees from across the world			
Annear et al. (31)		Vaccination of attendees Ensuring self-isolation and quarantine for new arrivals or symptomatic individuals, distributing personal protective equipment (e.g., facemasks) and promoting rigorous hand and respiratory hygiene	Restricting spectator attendance (creating a so-called event bubble), imposing social distancing rules, conducting mandatory diagnostic testing		Impose significant control measures
Miles et al. (32)					Implementing protective measures, including staffing, physical protection systems, perimeter control, access control, risk management, emergency management, crowd management, and traffic control; all form an integral part of international sport event management
Alshahrani et al. (12)	International travel increases the risk of transmitting communicable diseases across communities overcrowding, poor hygiene, and malnutrition (influenza)	– Vaccination with doses adjusted based on age and presence of comorbidities of the individual – Providing appropriate education to traveler's Pre-travel consultation on health and safety measures, including vaccination – Any defects in the previous plans must be discovered and processed, and the capacity of Qatar's hospitals and stockpiles should be increased due to the mass casualties that may occur. In addition to this, having an adequate workforce, providing appropriate training for the medical staff, and having multilingual services to address the language barrier is also essential – Travel precautionary measures including COVID-19 test certificates, quarantine, and digital apps			– Implementing an appropriate health surveillance system – Maintaining hand hygiene (washing and disinfecting), wearing a protective mask, and social distancing as preventive measures against COVID-19
Pshenichnaya et al. (9)	– High crowd densities, non-compliance with hygiene rules or inadequate sanitation may lead to enhanced transmission of infectious disease agents among attendees with a potential for globalization given the international component of the event – Non-compliance with basic hygiene rules, inadequate sanitation, and insufficient vaccination coverage	– Up to date with the routine vaccination courses recommended in their home country – Additional vaccines for those who may be at increased risk of a vaccine preventable illness due to their lifestyle choice, or pre-existing illness visit their travel medicine advisors prior to travel		Clinicians seeing ill-returned travelers	
Ahmed and Memish (33)	Lack of immunity due to non-vaccination		– Bio-surveillance to detect illnesses – Enact surveillance and disease reporting mechanisms during the mass gathering events themselves to be able to identify case clusters or even infectious disease outbreaks	– Countries must be ready with a public health surge capacity to respond to returning travelers – Control measures and means to prevent infections exported back by attendees to their countries of origin	– Collaborative approach – Sharing resources across sectors and agencies whether public or private entities is critical to mass gatherings being safe-guarded
Abubakar et al. (34)	– Existing influenza tests are inappropriate for prompt detection of all strains at mass gatherings – Risk factors for spread of infectious disease depend on setting, event, climate, likely mixing patterns, population attending the event, and possible infections	– Planning using a recognized framework depending on the nation involving a range of government and non-government agencies at local, regional, and national levels – All travelers to large events should be encouraged to visit a health-care provider 4–6 weeks before travel to manage any risk through vaccinations, drugs, and advice – Pre-event vaccination when appropriate, and vaccination of all individuals who are identified as not immunized previously	– Prompt isolation and treatment of detected infectious cases might have a role in preventing the spread of some infections – Continuous assessment of how the public health system, health-care system, and general community that are coping with increases in the number of cases of communicable diseases or disease risk related to the mass gathering. Risk assessment of communicable diseases should be both strategic and case based – Enhanced surveillance system during the event. Prompt recognition of emerging patterns of infectious diseases, using systems such as the WHO global alert and response system GeoSentinel and the EuroTravNet and other equivalents are functioning optimally rapid identification of an outbreak during an event		– Control measures, including vaccination adequate surveillance to identify the disease, appropriate respiratory hygiene – Prompt isolation and treatment of detected infectious cases might have a role in preventing the spread of some infections – Collaborative approach: all elements of planning (before, during, and after the event) require close liaison with international organizations, including recognition of the obligations of each nation state according – Robust routine surveillance system exists for likely pathogens – Adequate laboratory facilities are essential for the provision of accurate and timely confirmation or exclusion of individuals with the disease – Syndromic surveillance has been suggested as a composite approach to identification of disease syndromes, a process that usually needs to be complemented by appropriate laboratory surveillance – Required or recommended immunization and other health-care guidance
Gaines et al. (35)		– Education of travelers on preventive measures vaccination for hepatitis A & B, yellow fever, rabies (3 doses) – Pre-travel consultation 4–6 weeks before departure – Refer patients to a travel medicine specialist when needed – Before departure, travelers should contact their health insurance company to determine whether their policy includes coverage overseas and for emergency expenses such as aeromedical evacuation. Travelers are advised to consider supplemental travel health insurance with specific overseas coverage, including 24-h access to assistance for health care and medical evacuation contingency plans	Travelers who become sick or injured while traveling should seek immediate health care		
Wilson and Chen (13)	– Risk on travelers: locally endemic infections that may be unfamiliar to many travelers and clinicians (e.g., dengue, cutaneous larva migrans, malaria, yellow fever). Infections that may be more likely to occur because of crowding and activities related to the mass events. Non-communicable diseases and problems that stem from the high density of people engaged in competitive events in an environment that may be hot, volatile, or otherwise unstable – Risk on citizens: visitors also pose risks to the host country. Visitors could carry pathogens that could spark a local epidemic, if the local population is susceptible or local conditions favor spread Examples include a new influenza virus, a new coronavirus, or a new, virulent serogroup or strain of Neisseria meningitides piddly to travelers with potential exposure – Concerns might include a new genotype of dengue virus – Visitors who are only attending the mass sporting events (and are in urban areas) face fewer risks than those who will have more extended stays that include the Amazon basin and rural areas	Vaccination to influenza and measles			Enhanced surveillance will be important to identify infections early (Chikungunya virus)
Blumberg et al. (36)	With people coming to the country from many African countries, the risk of importing EVD existed, and required mitigation	– Vaccination of yellow fever as requirement for entry of travelers from endemic countries – Airport health staff screened incoming travelers for fever – A small medical facility was established at the airport for the isolation of patients – Extensive staff training was conducted using videos and demonstrations in the use of personal protective equipment (PPE) and infection control practices, as well as simulation exercises – Training sessions in the recognition and management of a range of communicable diseases were held for medical personnel	During the games, the Ministry of Health participated in daily all-hazard assessment with the National Organizing Committee and developed and shared daily situation reports		– While a strong national surveillance system supported by district outbreak response teams was already in place for epidemic-prone diseases, this was supplemented by a daily surveillance system for specific priority conditions pertinent to the event – A daily analysis attempted to establish trends. An emergency 24-h reporting system was established for persons with suspected meningitis or VHF, and for any outbreaks – An isolation facility was established in an existing health center outside of the major hospitals – The requisite export permits and transport arrangements were facilitated. The public health and hospital laboratories in Gaborone were able to test for malaria and meningitis and common pathogens – The Ministry of Health and Population of Congo was responsible for the overall coordination and delivery of health services, and worked in close collaboration with other ministries, the organizing committee, and the WHO, to ensure rapid detection and containment of infectious diseases, especially EVD – Enhanced surveillance for key notifiable diseases was implemented in all eleven stadia and other important locations like the airport
Wilson et al. (14)	Gengue: urban areas, Transmission is most intense during February through June influenza showed clear seasonality, peaking in June and July	– All travelers should be up to date on their routine vaccines •Hepatitis A •Influenza – Measles-mumps-rubella – Influenza and yellow fever – Advise travelers on specific risks – Examined and aggregated top diagnoses reported during June through September – Travelers should check their entry requirement with Brazilian authorities in their own countries as well as the Brazil Ministry of Health		Post-travel surveillance is important for infections with long incubation times	– Used the The GeoSentinel Surveillance Network that is an international network of specialized travel and tropical medicine clinics located on six continents – All sites collect data by using a standard reporting form on ill travelers seen during or after international travel – Anonymized data on demographics, travel history, reason for travel, pre-travel advice, hospitalization, major clinical symptoms, and final diagnoses assigned by the GeoSentinel site clinician are electronically entered into a central database – Diagnoses are selected from a standard list of >500 diagnostic codes and involve syndromic groupings alone if no etiology is defined or syndromic groupings plus specific etiologies where possible – All sites use the best reference diagnostic tests available in their own country – Country of exposure is identified by the clinician based on the travelers' itinerary, known endemicity patterns of the destinations visited, and incubation period of the illness
Wong et al. (15)	– Asymptomatic cases – As the virus was also found in stool samples, eight contaminated environments, such as soil, may pose a threat to outdoor sporting events infection-risky behaviors, such as spitting and touching the face, are common during football games	– Relevant health education and psychology supporting materials were provided for athletes – A work-from-home roster and flexible office hours have also been implemented for administrative staff, and they were instructed not to have direct contact with athletes – The HKSI strictly abided-by the government's policy on inbound travelers, all activities to Mainland China and mass activities in HKSI were suspended from February 8 onward	– To quantify these transmission-risky behaviors, we obtained video footage of four male professional football players with dedicated cameras for an entire match. We tracked their time of close body contact (defined as an inter-personal distance of <1.5 m) and frequency of infection-risky behaviors (touching the mouth, touching the eyes, touching the nose, and spitting) – Weekly updates to remind all personnel on personal and maintaining physical distance between individuals during and after training	– Upon the issuance of Government “Red Outbound Travel Alert,” all personnel returning to Hong Kong after March 5 from overseas must report their temperature and symptoms (if any) electronically for 14 days and optional COVID-19 tests were provided – All personnel returning from COVID-19 affected areas (even if not included the governments' compulsory quarantine regions) were required to self-isolate at home or a hotel for 14 days before returning to HKSI. All travel to the affected areas were disallowed during the corresponding period	– Minimize the number of people congregated at one single place and time through closed competitions with no spectators and minimizing non-essential personnel present at the venue, such as by canceling press conferences and interviews – Sporting equipment should be cleaned as frequently as possible – All personnel were required to measure body temperature and declare FTOCC (Fever, Travel, Occupation, Contact and Clustering) status before entering the institute and the daily body temperature report of all athletes were obtained
Duarte Muñoz and Meyer (37)	– The likelihood of respiratory disease transmission among members of a football team is not particularly large – One must not forget all the situations around training and competition which happen in dressing rooms, during social activities or during medical care	Vaccination guidelines should be strictly met	– Basic preventive measures among football players, coaches and staff members and the general public – Adequate hand hygiene and “coughing etiquette,” as well as abstaining from social gatherings, especially when symptomatic, are key – Among athletes it is also important to avoid sharing personal objects, such as towels and water bottles – Organizational measures to increase distance between dressing and showering athletes (e.g., use of more dressing rooms than usual) – Players should not be treated together in one room to avoid spread among medical personnel	Looking at ill travelers returned from Brazil who were subsequently seen at a GeoSentinel clinic	
Hoang and Gautret (38)	Most ill individuals with measles had not been vaccinated	Individual preventive measures such as cough etiquette, the use of face mask and disposable handkerchiefs and hand hygiene vaccination of measles, mumps, meningococcal Influenza, and pneumococcal diseases			
Vyklyuk et al. (39)	–		– Identify disease through a mobile application by detecting the tone and strength of the cough. The accuracy of this method of identification is 70% – Another method involves identifying infected people by fever – Measure body temperature through tools: thermometer, Stationary thermal imaging systems, thermal sensors, and mobile thermal imagers		
Gautret et al. (40)	Most illnesses among travelers attending the Olympics were linked to trave	Contributing to efforts to create enhanced international multidisciplinary surveillance			
Al-Romaihi et al. (41)	Prompt and effective response to CD outbreaks during MG events requires that frontline HCWs have the correct knowledge, adequate training, and proper attitude about CDs and outbreaks and especially those working in EDs (7, 8). It is also necessary for EDs to have the necessary preparedness to effectively and promptly respond to such drastic situations	– Getting HCWs and staff in hospitals ready and prepared for disasters in MG events: – Increase HCWs understanding of relevant concepts such as disasters, pandemics, and influenza – Trained in disaster-related subject			
McCloskey et al. (16)	–	The approach taken to the public health risk assessment was to follow the principles of risk analysis, surveillance and reporting, and response. In response to this risk assessment, systems were enhanced to provide additional surveillance data, improve understanding of the public health effect of the 2012 Games, and raise public awareness and understanding of public health concerns Authorities began public health planning more than 7 years before the Games, following the principles laid out in the WHO Communicable Disease Alert and Response for Mass Gatherings Guidelines, and the experiences of previous host cities Important to address public health issues with the utmost urgency The systems and capacity need to be in place to rapidly receive and analyze information from surveillance, reporting, and intelligence systems, and to identify and respond to any potential health protection threat	The national Center for Infectious Disease Surveillance and Control routinely collates reports of incidents, outbreaks, and adverse trends from across the UK; during the Games, in addition to undertaking this daily, they collated enhanced systems Daily analyses of mortality data were also done, and a new system was introduced for sentinel intensive care units to report unexplained illness of probable infectious cause This system involved clinicians in pediatric and adult intensive care units rapidly reporting cases using a customized web-based method During the 2012 Games a national event-based surveillance team was the hub for reporting of incidents and outbreaks of an infectious disease from across the UK that might substantially affect the Games, by their effect on venues, Olympic staff, athletes, or visitors, or by the public's perception of the Games The team enhanced established systems by reviewing and collating daily incident and response reports submitted by all local health-protection teams. The team also reviewed the national public health case-management system (HPZone) for incidents and diseases of special interest Information from both these sources was collated, and a Games-specific risk assessment made according to agreed criteria seven information about any notable events identified was routinely reported daily to the national coordination center, or more frequently, if needed		These systems were the HPA/NHS Direct Syndromic Surveillance System, which provides so-called pre-primary care data using call information from the health advice telephone service for a range of syndromes, and the HPA/QSurveillance National General Practitioner (GP) Surveillance System, one of the largest GP surveillance systems in Europe, which monitors weekly consultation data from a network of more than 3,500 GP practices across the UK For the first time syndromic surveillance reporting was undertaken at the Games polyclinic. This polyclinic, in the Athletes' Village in the main OlympicParalympic park, was the principal point of access to medical services for athletes and others. Medical facilities were also located in every sporting venue, as well as in one of the main hotels housing the OlympicParalympic family Each time a medical service was used, the doctor, first aider, physiotherapist, dentist, or other health-care provider recorded details of the consultation and treatment using a medical encounter form The HPA worked with international partners-particularly the European Center for Disease Prevention and Control (ECDC) and WHO-to set up enhanced international surveillance for the 2012 Games (34, 37). This international surveillance monitored and assessed the risk, on a day-to-day basis throughout the surveillance period, of any infectious disease threats abroad that had the potential to affect health in the UK, and, in particular, at the Games. The team undertook joint risk assessments of incidents identified as relevant through an agreed set of criteria designed for the Games, using methods developed for this purpose Enhanced clinical, public health, and environmental microbiology laboratory capability and capacity are necessary to meet the increased demands of a mass gathering. As well as additional routine testing requirements, response teams need the ability to rapidly scale up the testing capability as part of the response to an infectious-disease outbreak
McCloskey et al. (42)		Risk assessments for COVID-19 (panel) need to consider the capacity of host countries to diagnose and treat severe respiratory illness Encompassed joint planning, enhancement of health infrastructures, and taking proper pre-emptive and preventive measures to control infectious diseases on an international scale 1)General considerations at the beginning of the planning phase: • Risk assessment must be coordinated and integrated with the host country's national risk assessment • Comprehensive risk assessment (with input from public health authorities) reviewed and updated regularly (2)COVID-19 specific considerations: • Consult WHO's updated technical guidance on COVID-19 (3)Specific action plan for COVID-19: action plans should be developed to mitigate all risks identified in the assessment. Action plans should include: • Integration with national emergency planning and response plans for infectious diseases • Command and control arrangements • Any appropriate screening requirements for event participants • Disease surveillance and detection • Treatment • Decision trigger points (4) If the decision is made to proceed with a MG, the planning should consider measures to: • Detect and monitor event-related COVID-19 • Reduce the spread of the virus • Manage and treat all ill persons • Disseminate public health messages specific to COVID-19 (5) Risk communication and community engagement: • Event organizers should agree with the public health authority on how participants and the local population will be kept informed about the health situation, key developments, and any relevant advice and recommended actions	(6)Risk mitigation strategies: • Reducing the number of participants or changing the venue to prevent crowding, or having a participant-only event without spectators • Staggering arrivals and departures • Providing packaged refreshments instead of a buffet • Increasing the number of, and access to, handwashing stations • Promoting personal protective practices (hand hygiene, respiratory etiquette, staying home if ill) • Offering virtual or live-streamed activities • Changing the event program to reduce high-risk activities such as those that require physical contact between participants		
Murray et al. (43)		Developed new health and safety protocols before the July 24 start of the 2020 season. In addition, MLB made the decision that games would be played without spectators	– Mitigation strategies for COVID: 1. Minimize contact between players and staff members (tiers) 2. Symptom screening and testing 3. Isolation of persons testing positive and quarantine of close contacts 4. Face masks 5. Social distancing 6. Environmental cleaning and disinfection – Increasing cloth face mask use among players and staff members (i.e., at all times except on the field of play), limiting travel to essential staff members, and prohibiting visits to gatherings of large groups of persons Frequent diagnostic testing for rapid case identification, isolation of persons with positive test results, quarantine for close contacts, mask wearing, and social distancing		
Chan et al. (44)		Sport leagues was planned with a collaborative approach among key stakeholders, including public health authorities, other governmental agencies, AOSMA, AFL, and SANFL A COVID-19 protocol including physical distancing measures and regular COVID-19 testing was proposed with strict adherence required of officials, staff, players, coaches, and where necessary, members of their household. Targeted media and communications prior to the matches contributed to the management of expectations and motivations of the attending spectators Key messages of physical distancing, hygiene, and infection prevention and control measures were communicated to attending spectators both in the time leading up to the matches (*via* targeted communications as well as traditional and social media) Spectators were also discouraged to attend if displaying COVID-19 symptoms, required to provide accurate personal information for contact tracing and encouraged to download the COVID Safe application	Specifying number of attendees and increasing it on stages Physical distance between seats Reminders on preventive measures during the matches (*via* broadcasting of health campaigns and visual reminders including clear signage, ground markings, and visual overlays)		Early collaborative planning among key stakeholders, both from government agencies and non-government agencies
Aitsi-Selmi et al. (45)					For an evidence-based approach to the health impacts (including infectious disease control) of mass gatherings to be effective, it will be important to blend all-hazard risk management strategies across current global initiatives
Drury et al. (46)	Group identity, physical setting, norms, broader trends in public beliefs, and behaviors before and during the pandemic might affect crowd behaviors at live events (proximity behaviors)	A key objective of the communication strategy is to make the behaviors listed above into new norms: first, ensure that the venue is organized in such a way as to make desired behaviors (such as distancing) possible, second, draw on an understanding of the relevant group identity in order to promote the new norms (or rather, to promote new forms of behavioral expression for old social norms). Effective communication should stress the following messages about risk: unsafe behaviors put fellow group members at risk and not only within the venue; they also put everybody's families at risk and also the entire community at risk; this in turn would present a major risk to the standing of the group in the community. Third, it is important that messages address not only what group members should do (so-called “injunctive norms”), but also what they are typically doing (“descriptive norms”). Fourth, the source of information is as important as its content Designing pilot studies and evaluations of events to inform strategies for opening events with minimal risk of transmitting the virus	Preventive measures: physical distancing; wearing of face coverings; and regular handwashing or sanitizing Specific behaviors that are commonplace at live events-such as singing, shouting, chanting, hugging, jumping up and down-need to be limited or substituted. Many of the behaviors required, or that need to be limited, can be moderated by the environment in the venue: i) Limited access/density and effective management of the flow of people in and around the venue ii) Enforced wearing of face coverings (with special arrangements for those unable to wear them) iii) Hand-hygiene stations at multiple points in the venue iv) Minimal shared surfaces that require touching (e.g., contactless doors and lavatories).		
Al-Tawfiq et al. (47)		Utilizing the recommended vaccines	The use of masks, practice of social distancing, hand hygiene, and contact avoidance		
Dénes et al. (48)		To prevent imported epidemics, it should be emphasized that vaccinating travelers would most efficiently reduce the risk of epidemic, while requiring the minimum doses of vaccines as compared to other vaccination strategies			
Leal Neto et al. (49)					Participatory surveillance through community engagement is an innovative way to conduct epidemiological surveillance
Chiampas and Ibiebele (50)		Organizations need to have established scalable protocols for athletes who do contract the virus with symptom-based algorithms for length of time away from play and with screening for cardiac and pulmonary complications from COVID-19 encouraging our athletes to become immunized against the virus and educating our athletes about nutrition and the relation to immune health is important as we return to play			Hygiene and social distancing, use of masks, rigorous monitoring and screening of symptoms, widespread testing, comprehensive contact tracing, and considerations for travel and facilities
Hassanzadeh-Rad and Farzin (51)					Contact tracing: For audiences inside the stadiums, there should be obligatory rules for all federations in all countries to sell traceable electronic tickets for each seat. By doing this, if an infected patient with COVID-19 who has recently participated in a crowded sports match as a spectator is discovered in clinics or hospitals, it is feasible to track all seats in a certain distance from the infected patient's seat (e.g., seats located in a radius of 2 m from that seat) and by information provided with electronic ticket systems, targeted PCR testing (instead of blind testing or no testing) is performed for other at-risk audiences whose seats were in close vicinity of that of the infected patient
Eberhardt et al. (52)		The additional health risks of travelers to sporting events as the FIFA World Cup 2014 should be addressed in addition to addressing traditional health threats in pre-travel counseling			

### 3.4. Strategies followed during the event

During the sports events, numerous studies identified particular recommendations as shown in Table 3. These recommendations were basic preventive measures among football players, coaches, staff members, and the general public (37, 44, 46) including the use of masks (46, 47) and the practice of social/ physical distancing (29, 31, 46, 47) with adequate hand hygiene (37, 46, 47), coughing etiquette (37), and contact avoidance (37, 47). In addition, a couple of studies were conducted among athletes to avoid sharing personal objects such as towels and water bottles (37), test athletics activities during the events (28), send weekly updates to remind all personnel on maintaining physical distance between individuals during and after training (15), and implement the organizational measures to increase the distance between dressing and showering athletes (37). Also imposing social distancing rules (31) through the physical distance between seats, specifying the number of attendees (44), limiting access and effective management of the flow of the people in and around the venue (46), and limiting some behaviors at live events such as singing, hugging, and jumping (46).

Murray and McCloskey both reported in 2020 about risk mitigation strategies such as staggering arrivals and departure, offering virtual or live–streamed activities, increasing the number of and access to handwashing stations, reducing the number of participants, or having a participant-only event without spectators, symptoms screening and testing, frequent diagnostic testing for rapid case identification, isolation of persons with positive test results and quarantine for close contacts, limiting travel to essential staff members, and prohibiting visits to gatherings of large groups of persons (42, 43). In addition, a study recommended identification of the disease through a mobile application by detecting the tone and strength of the cough, in which the accuracy of it is 70%, or by checking fever (39). Furthermore, the ministry of health's participation with the National Organizing Committee in daily all-hazards assessment report about the situation (36), enhancement of established systems by reviewing and collating daily incident, outbreak, and response reports (16), daily analysis of mortality data by all local health-protection teams (16), development of enact surveillance and disease reporting mechanism in order to identify infectious disease outbreaks during the event (33, 34) and assessing the frequency of infection-risky behaviors such as spitting, coughing (15) are recommended strategies during the event (see Table 3 for summary of the strategies recommended during the event).

### 3.5. Post-event strategies

In general, post-event recommendations include post-travel surveillance, particularly for infections with long incubation times (14), and testing athletics activities post-event (28). Countries must be prepared with a public health surge capacity and implement control measures to prevent infections from being exported back to their countries of origin by attendees (33), and in a study conducted in Hong Kong 2020, all personnel returning to their nation were obliged to electronically record their temperature and symptoms for 14 days, and any staff arriving from a COVID-19 impacted country was self-isolated at home for 14 days (15) (see Table 3 for summary of the post-event strategies).

### 3.6. General recommendations

Several general recommendations to prevent the spread of infectious diseases were suggested in some studies as shown in Table 3. Infection control measures recommendations include hand hygiene, using a protective mask, and social distancing (12, 34, 50), prompt isolation, and treatment of detected infectious cases which aims to prevent the spread of infections (34), adequate vaccination and recommended immunization (34), strict monitoring and screening of symptoms, widespread testing (15, 50), and minimizing the number of people congregated at one single location through closed competitions with no spectators, as well as unnecessary personnel present at a venue (15). Furthermore, a couple of studies considered enhancing epidemic intelligence to detect incidents efficiently (8, 10), implementing standard operating procedures for epidemic response (10), and developing community mitigation plans as general recommendations to consider (29). Implementation of protective and control measures such as risk, emergency, and crowd management, physical protective systems (31, 32, 45), and contact tracing for the audience (50, 51) all form an integral part of any sports event management. Moreover, other studies assessed the establishment of an appropriate routine health surveillance system in order to identify infections early (12–14, 16, 34, 36, 49) such as Syndromic Surveillance (16, 34), a collaborative approach between the ministry of health and other ministries, the committee and WHO to ensure rapid detection and containment of infectious diseases (33, 34, 36, 44), and enhancement of clinical, public health, and environmental microbiology laboratory capability and capacity (16, 34).

## 4. Discussion

To the best of our knowledge, this is the first systematic attempt to comprehend communicable disease risk in the context of previous mass gatherings sport. Several key findings were highlighted including the risk factors of different infectious diseases and prevention strategies that were clustered into three stages: pre-, during, and post-sport event mass gatherings.

Traveling continues to pose risks related to the prolonged close contact with people who may be carrying transmissible illnesses (53). Traveling results in increasing mobility, overcrowding and localized high population density, which impact the transmission of infectious diseases among communities. Another important risk factor is crowd behavior. In contrast to our findings, it's found that developing common identities between crowds can transform hazardous mass gatherings into a health-promoting event (54). This is explained by how behaviors are influenced by group identity, physical setting, climate, and individuals participating (46).

The majority of studies included has identified pre-event recommendations. This indicates that pre-event recommendations are the first line of defense and, if implemented correctly, can reduce the risk of COVID-19 transmission. Supporting our findings, pre-event vaccination and travel medicine measures were successful in preventing epidemics of influenza A H1N1 during the worldwide pandemic in 2009 at the Hajj and the Asian Youth Games in Singapore (55, 56). Immunization is a critical component of travel medicine, particularly for high-profile events such as the World Cup. Additionally, employing health communication by generating and spreading educational materials in the press for the public. Similarly, a study included all of FIFA's risk management published articles from 1994 to 2011 showed the effectiveness of risk communication in any risk management strategy (57). They provided the residual risk levels associated with certain risk factors to stakeholders in an appropriate and accessible manner, allowing informed critical discussions (57). Communication and collaboration are crucial among public health authorities within the host country as well as across participants' home countries to ensure the spread of health information among visitors.

During-event recommendations normally receives the most attention when events take place. According to the findings of this scoping review, adequate respiratory hygiene measures and practices, as well as rapid responses from public health authorities to detect infectious cases, was found to lower the likelihood of outbreaks. Similar to these findings, a study done to evaluate the effect of preventive measures, including face masks, stadium capacity, and capacity proportion on the infection risk has found that with the introduction of face masks and hand washing methods, the infection risk was decreased by 86–95 percent (58). This demonstrates that violations of COVID-19 recommendations, including not wearing masks, social distance, and self-isolation, will have a considerable influence on the total number of COVID-19 cases, and other respiratory diseases.

Only few studies considered taking actions after the end of sports events, which is considered as a limitation in the preparedness process. It was found that post-travel infections become apparent soon after, with 43–79% of travelers becoming ill with a travel-related illness (59). In addition, global surveillance after the event can be used as a guide to detailed travel history during every patient encounter (60). A study conducted in Brazil found that skin problems, diarrhea, and febrile systemic infections are most prevalent in returned travelers (14). Similar to our findings, a study reported that despite the success with mitigating spread of diseases, the returning Saudi pilgrims who visited pilgrimage sites in Iran and Iraq were early sources of COVID-19 spread, contributing to 150,000 cases (61). Thus, applying post-travel surveillance would contribute to COVID-19 mitigation.

In addition, some general recommendations were suggested. These recommendations include increasing the speed and accuracy of existing surveillance capabilities, developing active surveillance systems, conducting a detailed risk assessment to prioritize infections, and having the capability to receive and evaluate data quickly are all diverse ways to improve surveillance during sports mass gatherings (62). Even though the enhancement of the surveillance system might be relevant to the sports event itself, it should benefit the host country's public health infrastructure eventually (63).

### 4.1. Implications and recommendations for practice represented in a model

**Figure 2:**
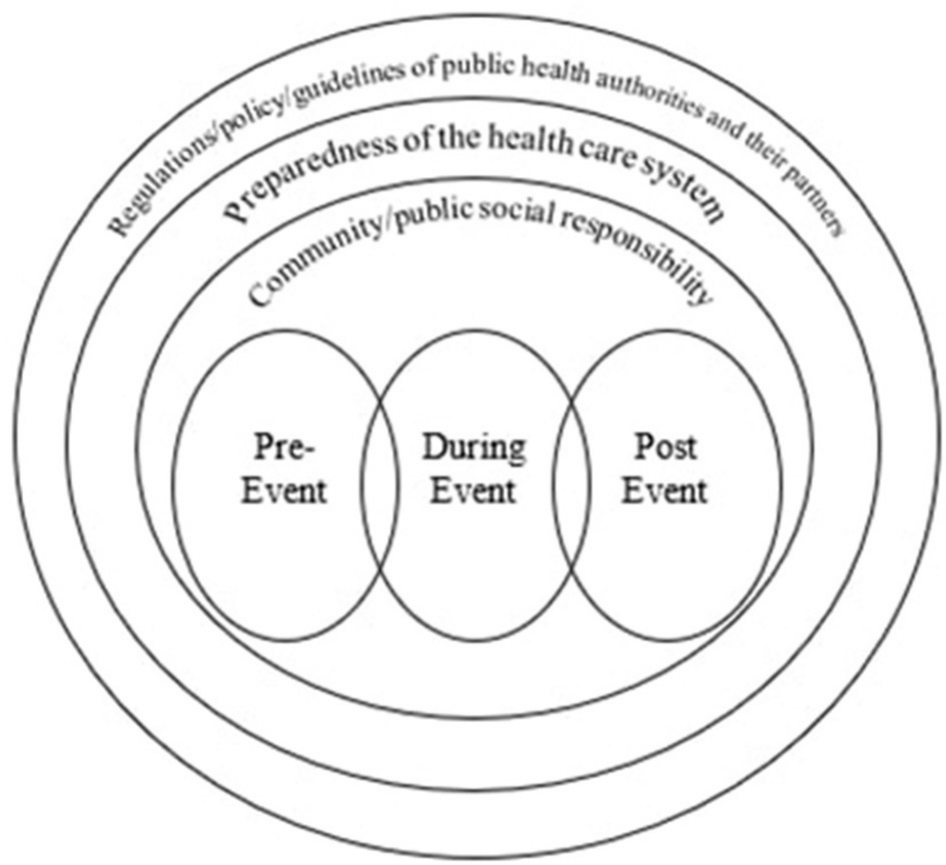
A model representing the three stages in which infectious disease prevention is followed during sport mass gatherings embedded in various contexts.

Based on this review, a model was constructed to represent the strategies followed to prevent infectious diseases in various stages of the event: pre-event, during the event, and post-event, and to describe the scope of literature. The model also shows how these strategies are related, and are supporting each other to achieve the goal of preventing infectious diseases during the three stages of the event. In addition, the model reflects on how these strategies are impeded in different three contexts that would support their implementation, monitoring, and evaluation. These contexts are the public social responsibility, preparedness of the health care system, and the regulations/policy/guidelines of public health authorities and their partners (see Figure 2).

It is recommended that tourists have a pre-travel consultation before traveling to consider suitable health and safety precautions, including vaccination. Since COVID-19 is still not over and there is a concern of emergence of new strains of SARS-CoV-2 virus, it is recommended that athletes, visitors, and citizens get the full vaccinations; provide them with a passport containing information about previous infections, results, testing, and vaccination status; and provide free rapid test centers for fans near each stadium, as well as directly matching these results in the spectators' passport. Authorities need to have an agreed preparedness plan, strengthen health emergency preparedness, and ensure the maintenance of precautionary measures for containing infectious diseases including COVID-19. Hospitals should be assessed with adequate workforce, providing appropriate training for the medical staff, and having multilingual services to address the language barrier are also essential. Event organizers should agree with the public health authority on how participants and the local people will be kept informed about the health situation, key developments, and any relevant recommended actions. Following these strategies would enhance the effective implementation of the other precautions during and after the effect.

Public health authorities and their partners authorities are advised to follow critical proposed recommendations, including implementation of the syndromic surveillance systems, or enhancing surveillance systems, disseminating public health messages specific to infectious diseases, and educating participants on prevention measures of these diseases. The public plays a vital role in mitigating any pandemic. Hence, enhancing social responsibility is a key to prevent outbreaks or combat the virus during events with mass gatherings. Low et al. illustrates that individuals' social responsibility actions are a result of the interaction between perceived infection risk and societal role responsibility. Public perception is critical in improving health risk communication, fostering public trust, and collaborating with the government's outbreak prevention efforts. Members of society can be empowered through organizations emphasizing their roles during the epidemic and recommend certain actions.

### 4.2. Implications for future research

This study will serve as a roadmap for preparedness for mega sports events in order to prevent infectious disease outbreaks. Our review reflects on a clear gap in quantitative evidence and highlights the need to conduct the quantitative assessment during the different stages of the event. Further observational research on post-sports mass gatherings is needed to explore various prevention strategies that should be implemented for this stage. In addition, it would be remarkably interesting to conduct qualitative research to study the perception of the public the World Cup hosting countries on social responsibility toward the World Cup, which may help improve future prevention and control efforts.

### 4.3. Strengths and limitations

This scoping review is the first to explore COVID-19 recommendations in the setting of the FIFA World Cup. The review includes a comprehensive search strategy and the most recent compilation of relevant up-to-date data from 2010 to January 24, 2022. There were no restrictions put on the study design, allowing for a broad exploration of peer-reviewed articles. This scoping review, however, has some limitations. A major limitation of this review is that the majority of the included studies are reviews and qualitative research, which reduces the quality of the evidence provided. Evidence of quantitative assessment is lacking in this scoping review and the quantitative contributions of the proposed specific recommendations in the prevention and control of infectious diseases cannot be certified, thus this is an urgent call for conducting quantitative research to provide evidence for effective planning for these events.

The included articles were not checked for validity in line with the scoping analysis approach, which is a less relevant method in scoping reviews. Furthermore, by excluding gray literature and non-English language literature, some bias may have been introduced.

## 5. Conclusion

The current scoping review identified a variety of studies and review articles that emphasize key findings, in order to develop a mitigation strategy for dealing with COVID-19 and other infectious diseases within the context of the FIFA World Cup The risk of COVID-19 infection and other infections among spectators at mass gathering events was reported. This review provides fundamental pre, during post-event recommendations to narrow and ideally achieve a “virus-free” event. The constructed model is reflecting on the importance of the involvement and empowerment of the public by enhancing their social responsibility and the coordination between the healthcare system, the ministry of public health, and other stakeholders for infectious disease prevention during the FIFA World Cup.

## Data availability statement

The original contributions presented in the study are included in the article/supplementary material, further inquiries can be directed to the corresponding author.

## Author contributions

NA, GA-J, and UE participated in creating the research questions, conceptualizing the idea of the project, building the search strategy, selecting studies, extracting data, analyzing data, and drafting and editing the manuscript. NH, MN, and MA participated in the data analysis, drafting, and editing the manuscript. All authors contributed to the article and approved the submitted version.
